# Guanine base modifications in antisense oligonucleotides mitigate acute central nervous system toxicity

**DOI:** 10.1039/d5cb00316d

**Published:** 2026-03-24

**Authors:** Maho Katsuyama, Taiki Matsubayashi, Yang Ying, Su Su Lei Mon, Takayuki Kuroda, Kie Yoshida-Tanaka, Rintaro Iwata Hara, Takeshi Yamada, Kumiko Ui-Tei, Kotaro Yoshioka

**Affiliations:** a Department of Neurology and Neurological Science, Graduate School of Medical and Dental Sciences, Institute of Science Tokyo 1-5-45 Yushima, Bunkyo-Ku Tokyo 113-8519 Japan kotanuro@md.isct.ac.jp; b Department of Neurology, National Hospital Organization Disaster Medical Center 3256 Midori-chou Tachikawa-shi Tokyo 190-0014 Japan; c Center for Brain Integration Research, Institute of Science Tokyo 1-5-45 Yushima, Bunkyo-ku, Tokyo 113-8519 Tokyo Japan; d NucleoTIDE and PepTIDE Drug Discovery Center, Institute of Science Tokyo 1-5-45 Yushima, Bunkyo-ku, Tokyo 113-8519 Tokyo Japan

## Abstract

Antisense oligonucleotides (ASOs) are recognized as promising therapeutic agents for central nervous system (CNS) diseases. However, neurotoxicity induced by ASOs *via* intrathecal administration poses a major limitation for clinical use. Several approaches have been reported to mitigate CNS toxicity; however, the effects of chemical modifications to nucleic acid bases on toxicity remain poorly understood. In this study, we investigated the effects of hypoxanthine substitution for other nucleobases in neurotoxic gapmer ASOs on toxicity and activity using *in vitro* and *in vivo* assays. Similarly, guanine modifications were evaluated for their influence on CNS toxicity and efficacy following intracerebroventricular injection in mice. We first found that substituting guanine with hypoxanthine mitigated neurotoxicity, whereas substituting adenine or cytosine with hypoxanthine exacerbated it. In contrast, all four types of hypoxanthine-substitutions reduced the binding affinity for target RNA and decreased *in vivo* silencing efficacy. We next identified several guanine modifications that alleviated neurotoxicity. In particular, 7-deazaguanine modification reduced CNS toxicity while maintaining the silencing activity of the ASOs. Our findings provide useful insights into nucleobase-dependent neurotoxicity and suggest a promising strategy involving guanine modifications to mitigate ASO-induced neurotoxicity without compromising therapeutic efficacy for the treatment of CNS diseases.

## Abstract 2

Guanine bases drive acute neurotoxicity of gapmer antisense oligonucleotides after intracerebroventricular administration, which is able to be mitigated by chemical modifications of guanines without compromising silencing activity.

## Graphical abstract

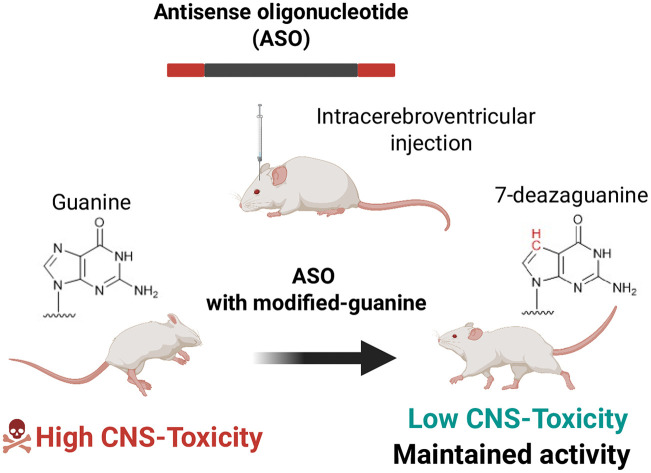

## Introduction

The clinical evidence demonstrating the efficacy and safety of antisense oligonucleotide (ASO) therapies is steadily increasing, accompanied by a growing number of drugs approved for clinical use.^1–3^ Because large, negatively charged molecules cannot cross the blood–brain barrier, ASOs are generally delivered to central nervous system (CNS) tissues *via* intrathecal administration.^4,5^ Intrathecal administration, in particular, is promising for CNS disorders that require long-term suppression of gene expression. Nusinersen, the first ASO drug approved for CNS application, was designed to alter target gene splicing and consists of a phosphorothioate (PS) backbone with fully 2′-*O*-methoxyethyl (2′-MOE) modifications.^6–8^ However, cases of aseptic meningitis have been documented after the initiation of nusinersen treatment.^9–11^ Likewise, tofersen, a gapmer ASO approved for the treatment of amyotrophic lateral sclerosis, has been associated with severe neurological adverse events—including myelitis, radiculitis, and aseptic meningitis—in clinical trials involving intrathecal administration.^12,13^ Thus, developing ASO therapeutics for CNS diseases requires not only potent gene-silencing but also expansion of the safety margin.

Gapmer ASOs bind target RNA in a sequence-specific manner to form ASO/RNA heteroduplexes, which are subsequently cleaved by RNase H. Chemical modifications such as locked nucleic acids (LNAs) in the wing regions enhance both binding affinity and resistance to nucleases, thereby improving therapeutic efficacy of ASO.^14,15^ Despite their therapeutic potential, several studies have reported behavioral abnormalities in the CNS following intrathecal injection of gapmer ASOs.^16–20^

In previous studies, sequence features of ASOs have been associated with neurotoxicity, with a shorter distance between the 3′ end and the guanine nucleotide correlating with increased acute toxicity, and moreover, guanine-rich sequences have been reported to exacerbate neurotoxic severity.^21,22^ In addition, T. Yoshida *et al.* reported that nucleobase modifications attenuate the hepatotoxicity of gapmer ASOs. These findings led us to hypothesize that nucleobase modifications could also mitigate neurotoxicity.^23^

In this study, we aimed to clarify the association between the neurotoxicity of LNA gapmers and the four types of bases in the gapmers. We then selected hypoxanthine as one of endogenous bases and investigated how substituting the four bases with the hypoxanthine affects neurotoxicity. Especially, we first focused on effect of guanine-substitution on neurotoxicity *in vitro* and *in vivo*, based on the previous studies reporting correlation between neurotoxicity and guanine.

In addition, we next investigated guanine chemical modifications to identify those that reduce toxicity without compromising ASO activity. Specifically, three guanine modifications, *N*^2^-methylguanine, *N*^2^-isobutylguanine, and 7-deazaguanine, were introduced. With respect to the *N*^2^ position, previous studies have demonstrated that chemical modification at the *N*^2^ position of guanine does not diminish the thermodynamic stability of the duplex.^24,25^ Accordingly, we hypothesized that the *N*^2^ position is one of potential modification site where the thermodynamic stability would remain largely unaffected. For 7-deazaguanine, previous studies have reported that the 7-deazaguanine substitution does not directly disrupt Watson–Crick base pairing,^26^ and that the modification does not decrease the *T*_m_.^23^

These ASOs were administered *via* the intracerebroventricular (i.c.v.) injection in mice, and acute neurotoxicity was evaluated using the acute tolerability scoring system (ATSS)^16,20,27^ and open-field test *in vivo*. In addition, gene-silencing efficacy was assessed in the mouse brain. Moreover, *T*_m_ measurements were performed to assess duplex formation between guanine-modified ASOs and complementary RNA.

Overall, we demonstrated a significant relationship between specific nucleotide bases and neurotoxicity. This finding was revealed through hypoxanthine substitution, whereby replacing guanine with hypoxanthine mitigates toxicity. Furthermore, we identified a guanine modification that simultaneously reduces neurotoxicity while maintaining therapeutic efficacy.

## Result

### Hypoxanthine substitution alleviates cytotoxicity of gapmer ASOs

**Fig. 1:**
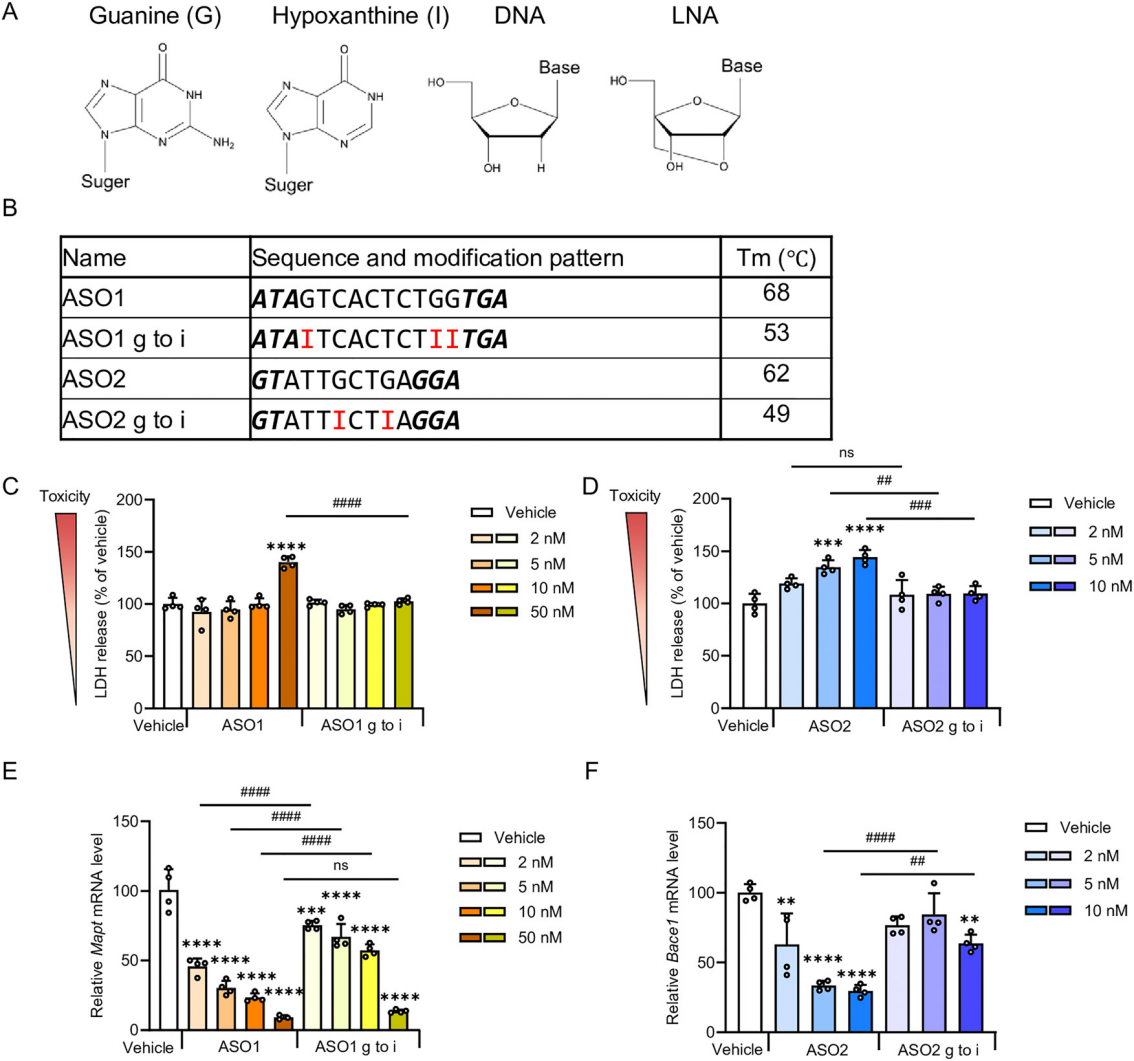
Effects of guanine-to-hypoxanthine substitution on ASO cytotoxicity and activity *in vitro*. (A) Chemical structures of guanine, hypoxanthine (base of inosine), DNA, and LNA, with LNA characterized by a 2′,4′-methylene linkage. (B) Sequence and *T*_m_ values of the LNA-gapmer ASO1 (16-mer) targeting mouse *Mapt* mRNA. (ASO1 g to i) represents sequences in which guanine have been substituted with hypoxanthine. Also shown are LNA-gapmer ASO2 (13-mer) targeting mouse *Bace1* mRNA and its hypoxanthine substituted derivative (ASO2 g to i). Capital letters indicate DNA, bold and italic letters indicate LNA-modified nucleoside, and all internucleoside linkages are PS bonds. G, A, T, C, and I represent DNA with guanine, adenine, thymine, cytosine, and hypoxanthine respectively. Hypoxanthine substitution sites are highlighted in red. (C) LDH release in Neuro-2a mouse neuroblastoma cells 48 h after transfection with 2, 5, 10, or 50 nM ASO1, expressed relative to vehicle. (D) LDH release in Neuro-2a cells 48 h after transfection with 2, 5, or 10 nM ASO2, expressed relative to vehicle. (E) Quantitative real-time PCR analysis of relative *Mapt* mRNA expression levels (% of vehicle) in Neuro-2a cells 48 h after transfection with 2, 5, 10, or 50 nM ASO1. (F) Quantitative real-time PCR analysis of relative *Bace1* mRNA expression levels (% of vehicle) in Neuro-2a cells 48 h after transfection with 2, 5, or 10 nM ASO2. Data are shown as mean ± SEM (*n* = 4 per group, C, D, F, *n* = 3–4 per group, E). Statistical differences were examined using a one-way ANOVA followed by a Tukey's *post hoc* test (*vs.* vehicle; *p < 0.05, **p < 0.01, ***p < 0.001, ****p < 0.0001) and comparisons between ASO and ASO g to i at the same concentrations (^#^*p* < 0.05, ^##^*p* < 0.01, ^###^*p* < 0.001, ^####^*p* < 0.0001). ns, not significant (*p* > 0.05).

To investigate binding affinity to target RNA, we first evaluated the *T*_m_ values of ASO1, ASO2 and their hypoxanthine-substituted derivatives. It is well established that substitution of guanine with hypoxanthine leads to a reduction in duplex stability, as reflected by a lower *T*_m_.^28,29^ Consistent with previous reports, our analysis revealed that hypoxanthine-substituted exhibited a decrease in *T*_m_ of more than 10 °C compared to the parental sequences (Fig. 1A and B).

We next evaluated the cytotoxicity of ASO1 and ASO2 as well as the effect of hypoxanthine substitution, Neuro-2a mouse neuroblastoma cells were transfected with ASOs, and LDH release together with cell viability was measured 48 h later. ASO1 induced a marked increase in LDH levels and tended to reduce cell viability at 50 nM (Fig. 1C and Fig. S1), whereas ASO2 elevated LDH levels in a dose-dependent manner, accompanied by a modest decline in viable cells (Fig. 1D and Fig. S1). In contrast, substitution of guanine with hypoxanthine in the gap regions of ASO1 and ASO2 significantly suppressed LDH elevation in both ASOs (Fig. 1C and D). In the cell viability analysis, hypoxanthine substitution in ASO1 and ASO2 tended to attenuate the reduction in cell number relative to original ASO1 and ASO2 (Fig. S1).

Analysis of target RNA expression confirmed concentration-dependent knockdown activity for both ASOs. In ASO1, hypoxanthine substitution decreased silencing activity at all concentrations except 50 nM, compared with the original ASO1 (Fig. 1E). In addition, a significant decrease in the intracellular efficacy of ASO2 was also caused by hypoxanthine substitution at concentrations of 5 nM or above (Fig. 1F). These findings suggest that hypoxanthine substitution reduces cytotoxicity but is accompanied by a decrease in *T*_m_, resulting in reduced intracellular activity.

### Guanine-to-hypoxanthine substitution mitigates acute CNS toxicity *in vivo*

**Fig. 2:**
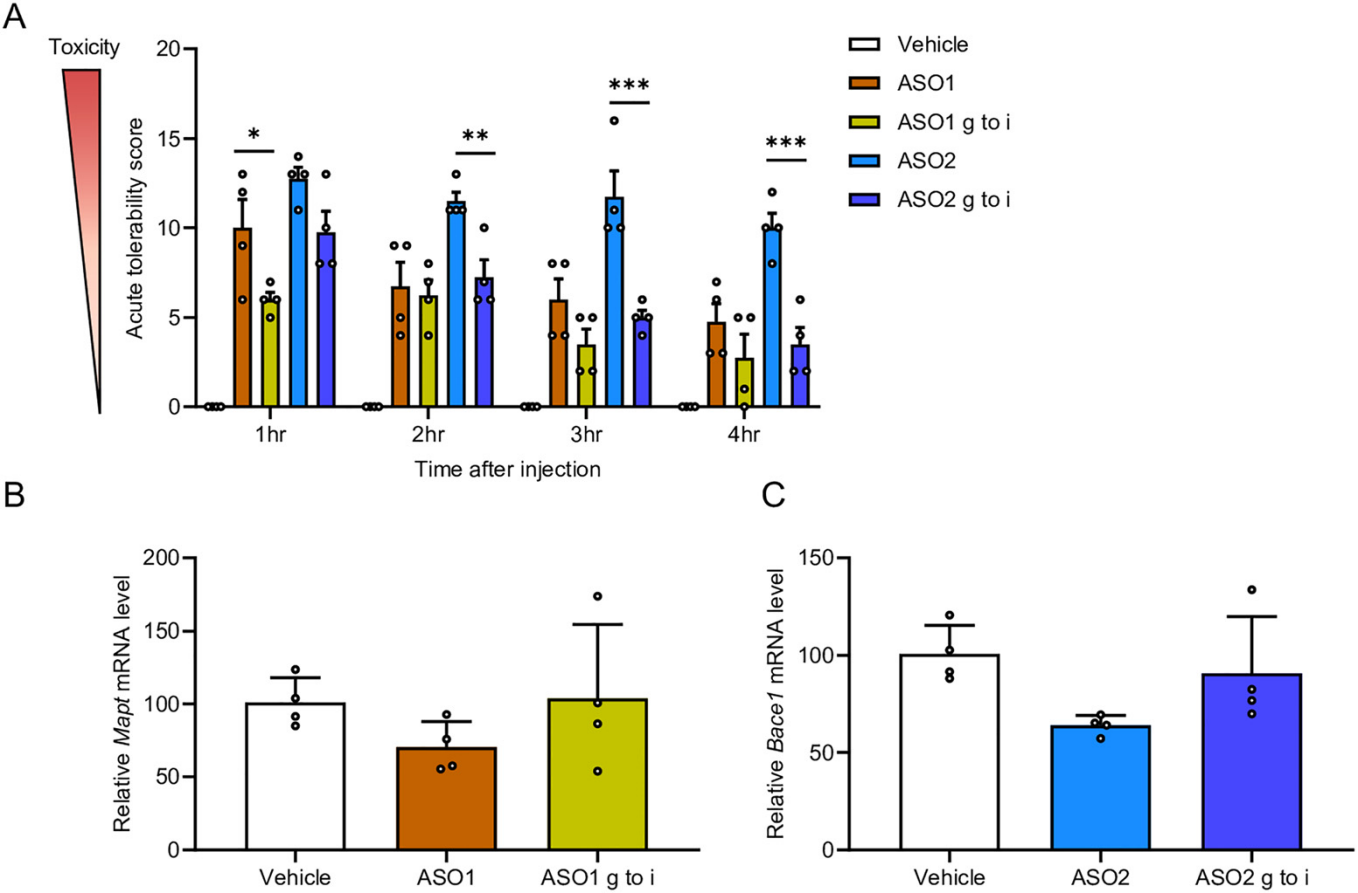
Effects of hypoxanthine substitution on acute neurotoxicity and silencing of target gene. (A) Acute tolerability scores in mice assessed 1–4 h after injection of ASO1, ASO1 g to i (9.4 nmol, 50 µg per mouse), ASO2, and ASO2 g to i (11 nmol, 50 µg per mouse). (B) Quantitative real-time PCR analysis of relative *Mapt* mRNA expression (% of vehicle) in the mouse hippocampus 7 days after i.c.v. injection. Data were analyzed using one-way ANOVA followed by Tukey's *post hoc* test (*vs.* vehicle). (C) Quantitative real-time PCR analysis of *Bace1* mRNA expression (% of vehicle) in the hippocampus. Data are shown as mean ± SEM (*n* = 4 per group, A–C). Statistical differences were evaluated using one-way ANOVA followed by Tukey's *post hoc* test (*p < 0.05, **p < 0.01, ***p < 0.001 *vs.* ASO1 or ASO2). No significant differences were observed where *p* > 0.05.

To investigate whether guanine-to-hypoxanthine substitution mitigates CNS-toxicity, ASO1 and ASO2 were administered i.c.v. to mice at doses of 9.4 nmol per head and 11 nmol per head, respectively (both equivalent to 50 µg per mouse). Neurobehavioral toxicity was evaluated with the ATSS (Table S1). The ATSS revealed that both ASO1 and ASO2 induced acute severe neurotoxicity beginning 1 h after i.c.v. injection. ASO1 with hypoxanthine substitution showed significantly lower neurotoxicity at the 1 h time point than the original ASO1 (Fig. 2A and Video S1). In addition, hypoxanthine substitution in ASO2 led to a significant improvement in neurotoxicity starting 2 h after i.c.v. injection (Fig. 2A). Conversely, target RNA expression analysis showed that hypoxanthine substitution in both ASO1 and ASO2 tended to decrease gene-silencing efficacy in the hippocampus (Fig. 2B and C).

Taken together, these findings indicate that the guanine to hypoxanthine substitution mitigates neurotoxicity in both *in vitro* and *in vivo* settings.

### Nucleobase-dependent effects of hypoxanthine substitution on *in vivo* CNS toxicity

**Fig. 3:**
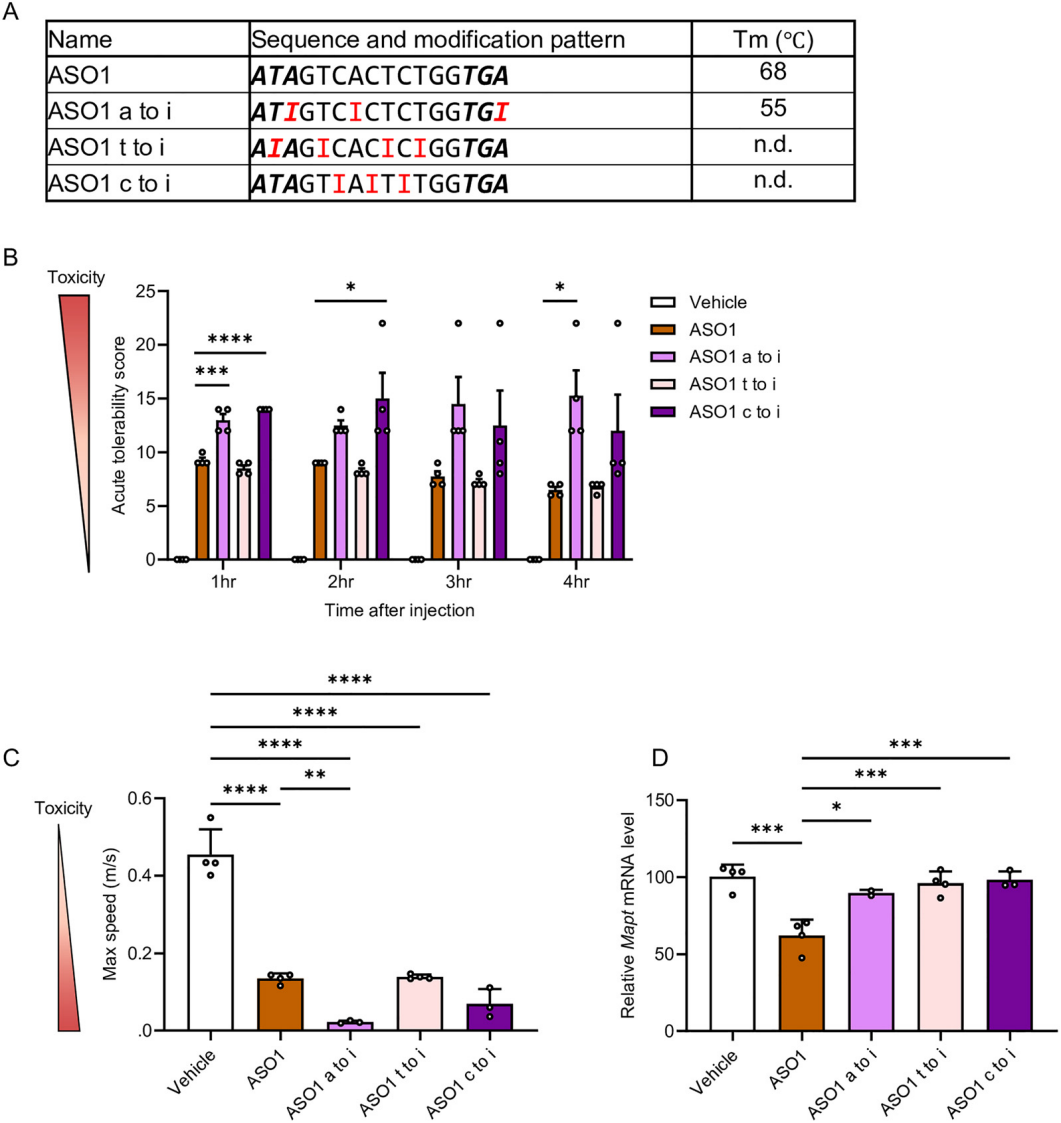
Comparative analysis of hypoxanthine substitutions at different bases reveals guanine-specific mitigation of acute neurotoxicity. (A) Sequences and *T*_m_ values of LNA-gapmer ASO1 (16-mer) targeting mouse *Mapt* mRNA. “n.d.” indicates values that were not determined. The terms “a to i,” “t to i,” and “c to i” refer to substitutions in which adenine, thymine, cytosine are respectively replaced by hypoxanthine. Capital letters indicate DNA, bold and italic letters indicate LNA-modified nucleoside, and G, A, T, C, and I represent DNA with guanine, adenine, thymine, cytosine, and hypoxanthine, respectively. (B) Acute tolerability scores in mice assessed 1–4 h after i.c.v. injection of ASO1, ASO1 a to i, t to i, and c to i (9.4 nmol, 50 µg per mouse). (C) Locomotor activity parameters (maximum speed) at 3 h post-injection in the open-field tests. Data are shown as mean ± SEM (*n* = 4), except for the ASO1 a to i group, in which one animal died 3 h post-injection (*n* = 3) and ASO1 c to i group, in which one animal died 2 h post-injection (*n* = 3). (D) Quantitative real-time PCR analysis of relative *Mapt* mRNA expression (% of vehicle) in the hippocampus. Data are presented as mean ± SEM (*n* = 4 per group), except for ASO1 a to i (*n* = 2, due to two animal deaths at 3–5 h post-i.c.v. injection) and ASO1 c to i (*n* = 3, due to one death at 2 h post-injection). Statistical differences were performed using one-way ANOVA followed by Tukey's *post hoc* test. (B): *vs.* ASO1 (*p < 0.05, **p < 0.01, ***p < 0.001, ****p < 0.0001). (C), (D): *vs.* vehicle and ASO1 (*p < 0.05, **p < 0.01, ***p < 0.001, ****p < 0.0001). No significant differences were observed where *p* > 0.05.

To further evaluate the base-specific effects of hypoxanthine substitution with other nucleobases, we tested whether replacing adenine, thymine, or cytosine with hypoxanthine in ASO1 altered CNS-toxicity *in vivo. T*_m_ analysis showed that substitution of adenine with hypoxanthine decreased *T*_m_ by more than 10 °C (Fig. 3A). In contrast, hypoxanthine substitution at thymine and cytosine resulted in duplexes that were not reliably measurable, owing to markedly reduced duplex stability (Fig. 3A).

These modified ASOs were administered *via* i.c.v. route at 9.4 nmol per head (50 µg per mouse), and neurotoxicity was assessed using ATSS and by analyzing locomotor activity in open-field test. Overall, substitution of adenine or cytosine with hypoxanthine increased ATSS scores relative to the original ASO1. In contrast, substitution of thymine with hypoxanthine did not alter ATSS scores (Fig. 3B and Video S2). Analysis of locomotor activity showed that substitution of adenine with hypoxanthine significantly reduced maximum speed compared with the original ASO1 at 3 h after i.c.v. injection (Fig. 3C). Substitution of cytosine with hypoxanthine tended to lower maximum speed at both 1 and 3 h relative to the original ASO1, whereas substitution of thymine with hypoxanthine did not affect maximum speed (Fig. 3C and Fig. S2).

Furthermore, silencing efficiency in the hippocampus was significantly reduced in all three groups of hypoxanthine-substituted ASOs at 7 days after injection (Fig. 3D), consistent with the decreased duplex stability observed in the *T*_m_ analysis.

Taken together, these findings indicate that the effect of hypoxanthine substitution on *in vivo* neurotoxicity depends on the type of nucleobase replaced.

### Guanine modifications mitigate *in vivo* CNS toxicity with ASO1

**Fig. 4:**
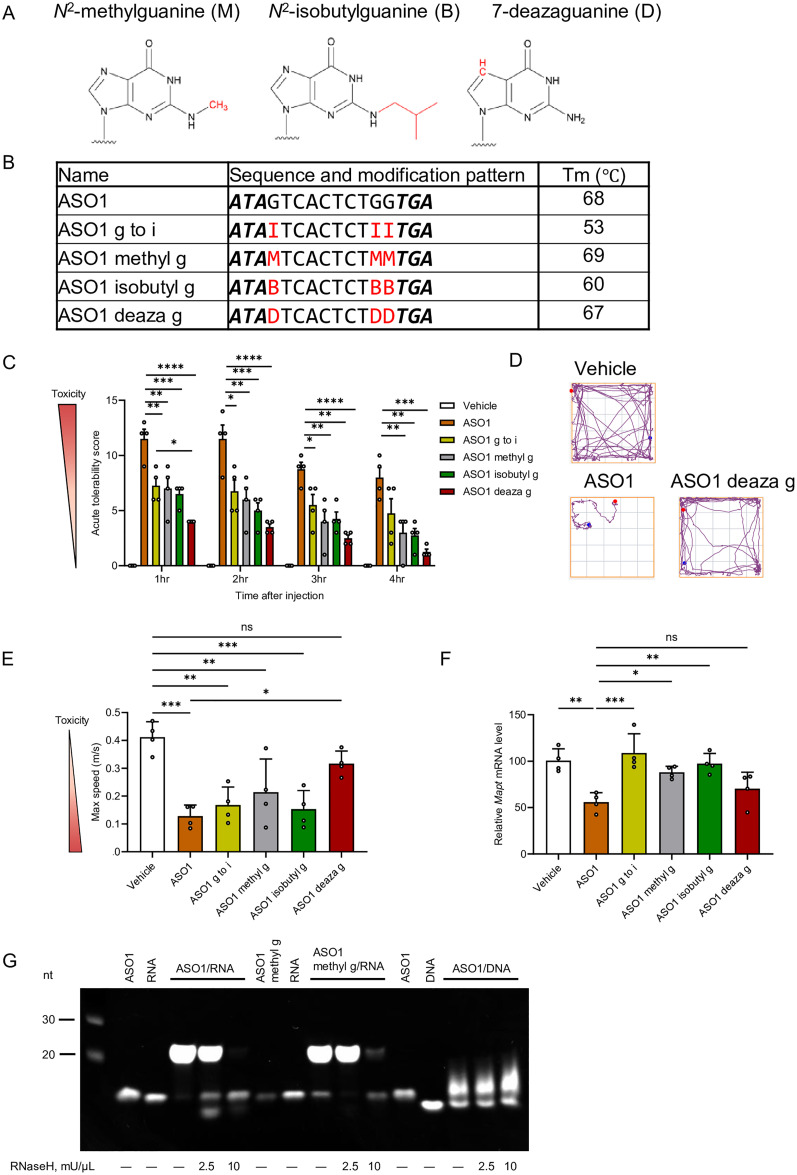
Guanine base modifications, particularly 7-deazaguanine, mitigate acute CNS toxicity of gapmer ASO1 targeting Mapt mRNA while maintaining silencing activity. (A) Chemical structures of *N*^2^-methylguanine, *N*^2^-isobutylguanine, and 7-deazaguanine. (B) Sequences and *T*_m_ values of LNA-gapmer ASO1 (16-mer) targeting mouse *Mapt* mRNA. Capital letters indicate DNA, bold and italic letters indicate LNA-modified nucleoside. All internucleoside linkages are phosphorothioate. G, A, T, C, and I represent DNA with guanine, adenine, thymine, cytosine, and hypoxanthine. ASO1 methyl g, ASO1 isobutyl g, and ASO1 deaza g represent guanine modified to *N*^2^-methylguanine, *N*^2^-isobutylguanine, or 7-deazaguanine, respectively. M, N, and D correspond to 2′-deoxyribonucleosides containing these modifications and are highlighted in red. (C) Acute tolerability scores in mice assessed 1–4 h after i.c.v. injection of ASO1, ASO1 g to i, ASO1 methyl g, ASO1 isobutyl g, and ASO1 deaza g (9.4 nmol, 50 µg per mouse). (D) Representative track plots from open-field tests conducted 3 h post-injection. (E) Locomotor activity parameters (maximum speed) assessed 3 h post-injection. (F) Quantitative real-time PCR analysis of relative *Mapt* mRNA expression in the hippocampus. (G) RNase H–mediated RNA cleavage activity. ASO1/RNA and ASO1 methyl g/RNA duplexes (1.25 µM) were incubated with *E. coli* RNase H (2.5 mU µL^−1^ or 10 mU µL^−1^) at 37 °C for 30 min. The reaction was stopped with stop solution and electrophoresed on a 15% denaturing PAGE gel. Gels were stained with GelRed and imaged using the ChemiDoc system. Data are presented as mean ± SEM (*n* = 4 per group for panels C, E, and F). Statistical analysis was performed using one-way ANOVA followed by Tukey's *post hoc* test. (C): *vs.* ASO-treated groups (*p < 0.05, **p < 0.01, ***p < 0.001, ****p < 0.0001). (E) and (F): *vs.* vehicle and ASO1 (*p < 0.05, **p < 0.01, ***p < 0.001).

As shown in previous experiments, Substitution of guanine with hypoxanthine mitigated neurotoxicity both *in vitro* and *in vivo*. We hypothesized that introducing new chemical modifications to guanine could further reduce neurotoxicity without diminishing silencing activity. To test this hypothesis, we introduced chemical modifications at the *N*^2^ or *N*^7^ guanine position (Fig. 4A). Specifically, three modified ASOs were synthesized by incorporating different guanine derivatives into the guanine within the gap region of ASO1: methyl g, containing *N*^2^-methylguanine; isobutyl g, containing *N*^2^-isobutylguanine; and deaza g, containing 7-deazaguanine. Consistent with previous reports demonstrating that *N*^2^-methylguanine and 7-deazaguanine do not significantly affect duplex stability,^24,30^ our analysis similarly showed that these modifications did not alter *T*_m_, whereas isobutyl g caused an 8 °C decrease (Fig. 4B).

These ASOs were administered i.c.v. to mice at a dose of 9.4 nmol per head (50 µg per mouse), and CNS-toxicity was evaluated. In ASO1, all three novel modifications significantly mitigated CNS toxicity compared to unmodified ASO1, as assessed by the ATSS, and also showed a tendency to improve locomotor activity (Fig. 4C–E and Fig. S3, Video S3). Notably, the deaza g modification attenuate neurotoxicity more effectively than the hypoxanthine substitution at the 1 h time point in the ATSS analysis (Fig. 4C).

Gene-silencing activity in the hippocampus, evaluated 7 days after ASO injection, showed that methyl g and isobutyl g modifications reduced silencing efficacy, whereas deaza g maintained knockdown efficiency comparable to that of unmodified ASO1 (Fig. 4F).

To assess whether the methyl g alters RNase H recognition and/or cleavage, we performed an *in vitro* cleavage assay using *E. coli* RNase H. After 30 minutes of reaction, the remaining full-length complementary RNA was more abundant in ASO1 with methyl g than in the original ASO1. This finding indicates that the methyl g modification in gapmer ASO reduces the RNase H activity compared to the unmodified guanine.

Overall, our findings suggest that the three types of guanine modifications mitigate *in vivo* CNS toxicity together with ASO1. Moreover, deaza g was associated with the highest mitigation effect without causing reduced silencing activity.

### Guanine modifications affect CNS toxicity *via* ASO2

**Fig. 5:**
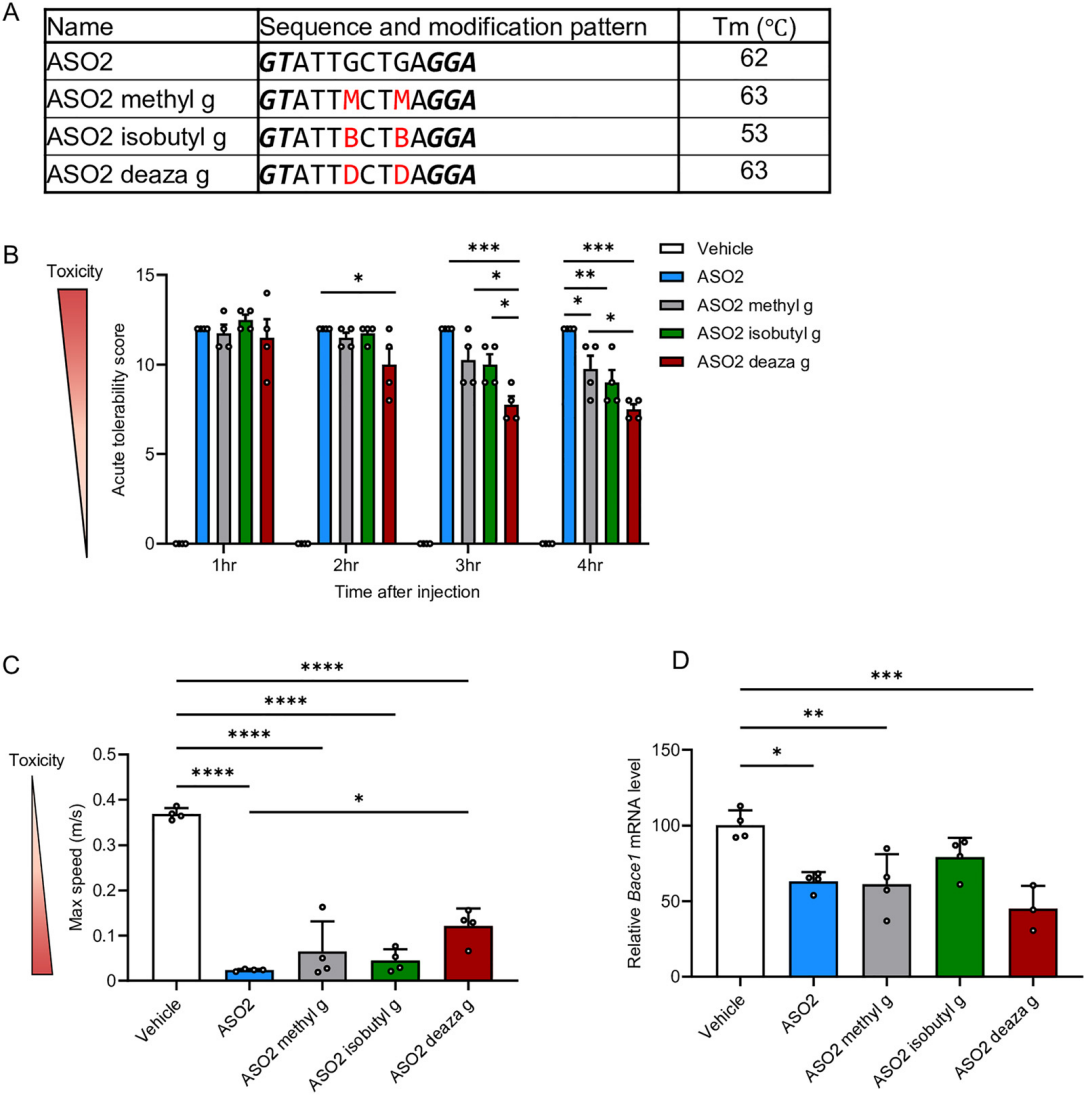
Guanine base modifications mitigate acute CNS toxicity of gapmer ASO2. (A) Sequences and *T*_m_ values of LNA-gapmer ASO2 (13-mer) targeting mouse *Bace1* mRNA. Capital letters indicate DNA, bold and italic letters indicate LNA-modified nucleoside. All internucleoside linkages are phosphorothioate. G, A, T, and C represent DNA with guanine, adenine, thymine, cytosine, respectively. ASO2 methyl g, ASO2 isobutyl g, and ASO2 deaza g represent guanine modified to *N*^2^-methylguanine, *N*^2^-isobutylguanine, or 7-deazaguanine, respectively. M, N, and D correspond to 2′-deoxyribonucleosides containing these modifications and are highlighted in red. (B) Acute tolerability scores in mice assessed 1–4 h after i.c.v. injection of ASO2, ASO2 methyl g, ASO2 isobutyl g, and ASO2 deaza g (11 nmol, 50 µg per mouse). (C) Locomotor activity parameters (maximum speed) assessed 3 h post-injection. (D) Quantitative real-time PCR analysis of relative *Bace1* mRNA expression in the hippocampus. Data shown indicate mean ± SEM (*n* = 4 per group, A–C). (D) One mouse in the ASO2 deaza g group died 7 days after administration, resulting in *n* = 3. Data were analyzed using one-way ANOVA followed by Tukey's *post hoc* test. (B); (*vs.* ASO-treated groups; *p < 0.05, **p < 0.01, ***p < 0.001). (C) and (D); (*vs.* vehicle and ASO2; *p < 0.05, **p < 0.01, ***p < 0.001, ****p < 0.0001). We observed no significant differences in group means (*p* > 0.05).

To determine whether the three guanine modifications could improve *in vivo* ASO-induced neurotoxicity beyond ASO1, we introduced the same guanine modifications into ASO2 and examined their potential to attenuate toxicity without reducing silencing efficacy (Fig. 5A).

In the *T*_m_ analysis, methyl g and deaza g did not decrease *T*_m_, whereas isobutyl g reduced it by 9 °C relative to the original ASO2; this finding is consistent with previous ASO1 results (Fig. 5A). Mice received i.c.v. administration of ASOs at 11 nmol per head (50 µg per mouse), after which CNS toxicity was evaluated. All three chemically modified ASOs showed significantly lower neurotoxicity than unmodified ASO2 at 4 h in ATSS analysis (Fig. 5B). In addition, deaza g showed the strongest mitigation effect among the three modifications from 2 to 4 h in ATSS analysis and 3 h in the open-field test (Fig. 5C and Fig. S4). Moreover, further evaluation of gene-silencing activity in the hippocampus revealed that ASO2 containing isobutyl g did not significantly reduce target RNA levels compared with the vehicle group, whereas ASO2 containing methyl g or deaza g, as well as the original ASO2, significantly decreased target RNA expression (Fig. 5D). These findings indicate that deaza g provides the greatest mitigation of *in vivo* neurotoxicity among the three modifications, while maintaining gene-silencing efficacy comparable to that of parental ASO2.

## Discussion

Our data demonstrate that substituting guanine with hypoxanthine improved neurotoxicity outcomes, both *in vitro* and *in vivo*. Furthermore, chemical modification of guanine also reduced *in vivo* neurotoxicity, with 7-deazaguanine showing the most pronounced effect. In contrast, hypoxanthine substitution diminished intracellular activity, whereas 7-deazaguanine maintained activity comparable to that of the parent ASO.

In our study, guanine-to-hypoxanthine substitution significantly suppressed LDH release in both ASO1 and ASO2 (Fig. 1), indicating that hypoxanthine contributes to attenuation of ASO-induced cytotoxicity. Because hypoxanthine has been reported to mimic guanine in its interactions with standard protein side chains,^31^ the substitution of guanine with hypoxanthine may attenuate cytotoxicity by altering interactions with proteins involved in cytotoxic responses.

The guanine-to-hypoxanthine substitution mitigated neurotoxicity *in vivo* (Fig. 2). This result suggests that guanine plays a critical role in inducing neurotoxicity, consistent with previous studies reporting that guanosine exacerbates acute neurotoxic outcomes.^21,22^ Hagedorn *et al.* reported a correlation between the severity of acute neurotoxicity and reduced α-amino-3-hydroxy-5-methyl-4-isoxazolepropionic acid (AMPA) receptors dependent spontaneous calcium oscillations in primary neurons, and further showed that ASOs with higher guanine content had lower calcium oscillation scores.^21^ In addition, we previously reported that a decrease in intracellular free calcium concentrations in primary rat neurons was associated with the severity of acute neurotoxicity *in vivo*, suggesting that inhibition of AMPA receptor function by ASOs may be one of the potential mechanisms underlying calcium-dependent toxicity.^27^ Guanine nucleotides are known to act as competitive inhibitors at AMPA receptors.^32–35^ Based on these previous findings, the reduction of the neurotoxicity with our guanine-to-hypoxanthine substitution indicates that neurotoxicity of ASOs is mainly caused by non-specific interactions of guanine with cell-surface proteins, including AMPA receptors.

By contrast, substitution of thymine with hypoxanthine did not alter neurotoxicity, and substitution of adenine or cytosine with hypoxanthine markedly exacerbated it (Fig. 3). O’Rourke *et al.* reported that cytosine- and adenine-rich sequences were associated with improvement of neurological impairment, including sedation, whereas thymine-rich sequences exerted no specific effects on neurological impairment.^22^ Our results are aligned with these findings.

Our findings showed that two chemical modifications of guanine, namely *N*^2^-methylguanine and *N*^2^-isobutylguanine, attenuated the acute neurotoxicity of the parental ASOs. Moreover, 7-deazaguanine substantially reduced the toxicity of both ASO1 and ASO2 (Fig. 4 and 5). Previous studies have reported that a decrease in the number of PS modifications in the ASO backbone improves CNS tolerability.^17,27^ In addition, other research indicates that reducing of PS modifications has shown to decrease non-specific protein binding.^17,36,37^ Based on these findings, our guanine modifications may affect binding to cell-surface proteins, including AMPA receptors, resulting in the mitigation of neurotoxicity.

In our *in vivo* analysis, substitution of guanine, adenine, thymine, or cytosine with hypoxanthine at gap positions resulted in reduction of silencing activity with the original gapmer-ASO. Based on our *T*_m_ analysis, all hypoxanthine-substituted ASOs resulted in a decrease in *T*_m_ by more than 10 °C across all ASOs tested (Fig. 2 and 3), which may be caused by the reduced hydrogen bonding of I–C pairs and formation of mismatches between hypoxanthine and other bases. The reduction of silencing activity can be explained by the decrease in *T*_m_.

Importantly, the 7-deazaguanine modification in ASO1 not only reduced toxicity but also maintained gene-silencing activity, whereas the hypoxanthine, *N*^2^-methylguanine, and *N*^2^-isobutylguanine modifications diminished this activity. Previous studies have reported that hypoxanthine destabilizes Watson–Crick base pairing with cytosine, but 7-deazaguanine does not.^26,38–41^ This suggests that 7-deazaguanine has a smaller impact on target RNA hybridization than hypoxanthine, which is corroborated by the observation in this study that 7-deazaguanine modification maintained the *T*_m_ of ASO1. Methylguanine modifications also maintained the *T*_m_, but they diminished the gene silencing activity. *In vitro* cleavage assays using RNase H indicated that *N*^2^-methylguanine modification reduced the cleavage efficiency of RNase H compared with unmodified guanine (Fig. 4). Therefore, it is possible that the methylguanine modification altered the interaction with RNase H, resulting in a decrease in activity. A previous study reported that some bulky substituents at the *N*^2^ position of guanine altered the recognition and cleavage pattern of RNase H and the alteration was expected to result from steric hindrance with the RNase H.^42^ Based on this report, the reduced knockdown activity with the ASOs with *N*^2^-isobutylguanine can be explained by impairment of recognition or cleavage with RNase H, which may be caused by steric hindrance due to the bulky structure at the *N*^2^ position in *N*^2^-isobutylguanine. There are some limitations in this study. Further studies are required to elucidate the mechanisms underlying the changes in efficacy and the toxicity in our study because the potential contributions of AMPA receptor interactions and alterations in RNase H binding affinity remain unclear. Moreover, future studies should assess whether these modifications influence off-target effects. In addition, while this study focused on mitigation of the neurotoxicity with the LNA-gapmers, future investigations that evaluate whether hypoxanthine substitution or guanine modification provides similar toxicity-reducing benefits across ASOs of different lengths and chemical modifications will be informative for designing new therapeutic approaches. Finally, although mitigation of neurotoxicity has been demonstrated following i.c.v. administration, whether comparable outcomes can be achieved through intrathecal delivery remains unclear. Addressing this question will provide important insights into the safety improvements conferred by guanine modifications.

Taken together, these findings highlight the potential of nucleobase modifications as a novel approach to improve CNS safety.

## Conclusions

In this study, we demonstrated that chemical modification of guanine is an effective strategy to reduce the acute neurotoxicity of LNA-gapmer ASOs. While previous reports have shown that a reduction in PS linkages, and sugar modifications, including 2′-*O*-methyl, 2′-MOE, and 5′-cyclopropylene alleviate neurotoxicity,^17,20–22,27^ our findings highlight the potential of base modifications to reduce neurotoxicity. Among them, 7-deazaguanine is particularly promising because it reduces toxicity while maintaining silencing activity. This strategy offers a new direction for ASO design targeting CNS diseases and represents an important step toward improved safety.

## Material and methods

### ASO design and synthesis

This study employed two DNA/LNA gapmer-type ASOs with PS backbone, previously reported by our group to induce toxicity *in vivo*.^27^ ASO1 targets mouse microtubule-associated protein tau (*Mapt*) mRNA, and ASO2 targets the mRNA of β-site amyloid precursor protein cleaving enzyme 1 (*Bace1*).^43^ All ASOs, including the original sequences as well as those containing hypoxanthine substitutions and guanine chemical modifications (*N*^2^-methylguanine, *N*^2^-isobutylguanine, and 7-deazaguanine), were synthesized by Gene Design (Osaka, Japan) and prepared in 1× phosphate-buffered saline (PBS; Nacalai Tesque, Tokyo, Japan).

### Cell culture conditions and ASO transfection

We cultured a Neuro-2a mouse neuroblastoma cell line in DMEM (Wako, Osaka, Japan) supplemented with 10% fetal bovine serum (FBS) and 1% penicillin/streptomycin. Culturing was performed in a humidified incubator at 37 °C with 5% CO_2_. Cells (3 × 10^4^) were seeded into 48-well plates and transfected with increasing ASO concentrations using Gibco Opti-MEM medium (Thermo Fisher Scientific, Waltham, MA, USA) containing 5 µg mL^−1^ Lipofectamine 2000 (Invitrogen, Carlsbad, CA, USA). PBS was used as the vehicle for the negative control. After 3 h of incubation, DMEM supplemented with FBS and penicillin/streptomycin was added to the transfection mixture. After this addition, cells were incubated for another 48 h at 37 °C with 5% CO_2_ for later experiments.

### Cytotoxicity assays

Lactate dehydrogenase (LDH) activity in the culture supernatant was measured using the Cytotoxicity LDH Assay Kit-WST (Dojindo, Kumamoto, Japan). Cell metabolic activity, as an indicator of viability, was evaluated with the Cell Counting Kit-8 (Dojindo, Kumamoto, Japan). Absorbance was recorded using a TECAN M1000Pro microplate reader (Tecan, Mänedorf, Switzerland).

### Animals and i.c.v. injection

We used wild-type female Crlj: CD1 (ICR) mice at 7 weeks of age for all experiments. Animals were obtained from Oriental Yeast Co., Ltd. or Sankyo Labo Service Corporation (Tokyo, Japan).

For i.c.v. bolus injection, mice were first anesthetized with 2.5% isoflurane for induction then maintained under 2.0% isoflurane during the procedure. Each mouse was secured to a stereotaxic frame (Narishige, Tokyo, Japan) using a head holder adapter and auxiliary ear bars. After a midline scalp incision and skull exposure, a burr hole (0.3 mm in diameter) was drilled 1 mm lateral to the midline and 0.2 mm posterior to the bregma. We then mounted a 25 µL glass syringe fitted with a 33-gauge needle (Hamilton Company, Bonaduz, Switzerland) on a motorized stereotaxic microinjector (IMS-30). The needle was slowly inserted into the left lateral ventricle *via* the burr hole. ASO solutions in PBS or PBS alone (*i.e.*, a vehicle control) were injected at a rate of 15 µL over 3 min (*n* = 4 per group). 5 min after injection, the needle was slowly withdrawn, and the scalp was closed with nylon sutures.

All animal procedures complied with institutional guidelines for the safe and ethical use of laboratory animals. All experimental procedures were approved by the Institutional Animal Care and Use Committee of the Institute of Science Tokyo (Approval No. A2025-125A).

### Acute toxicity evaluation using ATSS

To assess abnormal neurological behaviors, mice were observed both in their home cages and outside; this permitted evaluation of muscle strength and righting reflex. Acute neurotoxicity was scored using the ATSS, a previously established evaluation method comprising five categories:^16,20,27^ (1) consciousness, (2) motor function, (3) appearance, (4) hyperactivity, and (5) involuntary movement (Table S1). Each category was rated on a 5-point scale, and the acute tolerability score was the sum of all categories (minimum = 0, maximum = 20). A score of 22 was assigned to mice that died during or after i.c.v. administration. To ensure reliability, scoring was performed by a second evaluator who was blind to treatment; scoring was performed at 1, 2, 3, and 4 h post-injection.

### Open-field test

Each mouse was placed at the center of a square open-field arena (50 cm × 50 cm × 40 cm; Muromachi Kikai Co., Tokyo, Japan) consisting of a floor and surrounding walls, and allowed to explore freely for 5 min. Behavior was recorded using an automated video tracking system (ANY-maze, Stoelting Co., IL, USA). Locomotor parameters, including total distance travel and maximum speed were analyzed at 1 and 3 h after i.c.v. injection.

### RNA isolation and quantitative real-time PCR assay

Total RNA was extracted from *in vitro* and mouse brain samples using ISOGEN I (Nippon Gene, Tokyo, Japan) following the manufacturer's instructions. Complementary DNA (cDNA) was synthesized from the isolated RNA with the Takara 5× PrimeScript RT Master Mix (Takara Bio Inc., Kusatsu, Shiga, Japan). Quantitative real-time PCR was carried out with the LightCycler 480 Probes Master kit (Roche Applied Science, Penzberg, Germany) on a LightCycler 480 system (Roche Diagnostics, Mannheim, Germany). Primers and probes for mouse *Bace1* and *Mapt* were designed by TaqMan (Applied Biosystems, Foster City, CA, USA), while those for *Actb* were designed by Sigma-Aldrich (St. Louis, MO, USA). All qPCR experiments adhered to the Minimum Information for Publication of Quantitative Real-Time PCR Experiments (MIQE) guidelines.^44^

### *T*_m_ analysis

ASOs were then annealed with complementary RNA by heating to 95 °C for 5 min, followed by gradual cooling to room temperature. Duplexes were then diluted in PBS to a final concentration of 2 µM. Melting curves were generated by recording absorbance at 260 nm as samples were heated from 20 °C to 95 °C at a rate of 0.5 °C per minute. *T*_m_ values were determined from the first derivative of the absorbance curves. The *T*_m_ analysis of ASOs was analyzed by monitoring UV absorbance using the TMSPC-8 temperature-controlled accessory (Shimadzu, Kyoto, Japan).

### RNase H1 cleavage assays

The ASO and its complementary RNA were annealed and adjusted to a final concentration of 1.25 µM in a buffer containing 50 mM Tris–HCl and 7 mM KCl. Next, a 20 µL enzyme solution containing 50 mM Tris–HCl (pH 8.0), 75 mM KCl, 50 mM MgCl_2_, 5 mM DTT, and either 2.5 mU µL^−1^ or 10 mU µL^−1^ RNase H (2 U µL^−1^, *E. coli*, Invitrogen™, Thermo Fisher Scientific, USA) was added to the duplex solution, resulting in a final reaction volume of 100 µL. The reaction mixture was incubated at 37 °C for 30 min.

A 10 µL aliquot of the reaction mixture was collected, and the reaction was stopped by adding 10 µL of a stop solution consisting of 10 M urea, 50 mM EDTA·2Na, and 0.1 wt% bromophenol blue. Finally, 12 µL of each sample was loaded onto a 15% denaturing polyacrylamide gel (SuperSep™ RNA 15% gel(17-well), FUJIFILM Wako Pure Chemical Corporation, Japan) and electrophoresed at 200 V for 45 min at room temperature. A single stranded RNA ladder (20–100 nt) was used as a size marker. The gel was stained with GelRed (1:10,000 dilution) for 10 min with gentle shaking at room temperature, followed by imaging using a ChemiDoc Imaging System.

### Statistical analyses

All data were analyzed using GraphPad Prism version 10.1.2 for Windows (GraphPad Software, San Diego, CA, USA). Both *in vivo* and *in vitro* datasets were evaluated by one-way analysis of variance (ANOVA), followed by Tukey's *post hoc* multiple comparison tests, as appropriate. For all statistical tests we report *p*-values or “ns” (*i.e.*, to indicate that there was no statistically significant difference between group means.) Statistical significance was defined as *p* < 0.05. All graphs present mean ± SEM (standard error of the mean).

## Author contributions

M. K.: investigation, data analysis, visualization, data curation and writing the original draft. T. M.: conceptualization, data analysis, investigation and methodology. Y. Y.: investigation, formal analysis and methodology. S. S. L. M.: investigation, methodology and resources. T. K.: investigation. K. Y. T.: investigation and resources. R. I. H.: conceptualization and supervision. T. Y.: conceptualization and supervision. K. U. T.: supervision. K. Y.: conceptualization, funding acquisition, project administration, supervision, methodology and mentored the research. All authors contributed to review & editing of the manuscript and supplementary information (SI).

## Conflicts of interest

There are no conflicts to declare.

## Supplementary Material

CB-007-D5CB00316D-s001

CB-007-D5CB00316D-s002

CB-007-D5CB00316D-s003

CB-007-D5CB00316D-s004

CB-007-D5CB00316D-s005

CB-007-D5CB00316D-s006

CB-007-D5CB00316D-s007

CB-007-D5CB00316D-s008

CB-007-D5CB00316D-s009

CB-007-D5CB00316D-s010

CB-007-D5CB00316D-s011

CB-007-D5CB00316D-s012

CB-007-D5CB00316D-s013

CB-007-D5CB00316D-s014

## Data Availability

The data supporting this article have been included as part of the supplementary information (SI). Supplementary information: details on the acute tolerability scoring system, cytotoxicity assays, and the evaluation of spontaneous behaviors in mice. See DOI: https://doi.org/10.1039/d5cb00316d.
