# Supplementary material for: Guanine base modifications in antisense oligonucleotides mitigate acute central nervous system toxicity
Source: RSC Chem Biol. 2026 Mar 24;7(5):851–61. doi: 10.1039/d5cb00316d (PMC13040562; doi:10.1039/d5cb00316d)
Supplement: CB-007-D5CB00316D-s001 [file CB-007-D5CB00316D-s001.pdf]

Supplementary Information (SI) for RSC Chemical Biology.
This journal is © The Royal Society of Chemistry 2026

Supplementary information for

Guanine Base Modifications in Antisense Oligonucleotides Mitigate Acute CNS Toxicity

Maho Katsuyama [a, c-e], Taiki Matsubayashi [a-e], Yang Ying [a], Su Su Lei Mon [a,c,d], Takayuki Kuroda [a,c,d], Kie Yoshida-Tanaka [a,c,d], Rintaro Iwata Hara [a,c,d], Takeshi Yamada [a,c,d], Kumiko Ui-Tei [c,d], Kotaro Yoshioka [a,c,d]*

AFFILIATIONS

[a] Department of Neurology and Neurological Science, Graduate School of Medical and Dental Sciences, Institute of Science Tokyo, 1-5-45 Yushima, Bunkyo-Ku, Tokyo, 113-8519, Japan

[b] Department of Neurology, National Hospital Organization Disaster Medical Center, 3256 Midori-chou Tachikawa-shi, Tokyo 190-0014, Japan

[c] Center for Brain Integration Research, Institute of Science Tokyo, 1-5-45 Yushima, Bunkyo-ku, Tokyo, 113-8519 Tokyo, Japan

[d] NucleoTIDE and PepTIDE Drug Discovery Center, Institute of Science Tokyo, 1-5-45 Yushima, Bunkyo-ku, Tokyo, 113-8519 Tokyo, Japan

[e] These authors contributed equally

A

<table>
<tr><th></th><th></th><th>Score 1</th><th>Score 2</th><th>Score 3</th><th>Score 4</th></tr>
<tr><td rowspan="2">Category 1
Consciousness</td><td>Decreased exploration or voluntary movement</td><td>Decreased exploration</td><td>Decreased exploration</td><td>No exploration</td><td>No exploration</td></tr>
<tr><td>Decreased responsiveness</td><td>Normal</td><td>Slightly (e.g., reduced response to touch or handling)</td><td>Moderately (e.g., reduced response to lift, no blink)</td><td>Marked (e.g., reduced response to a tail pinch)</td></tr>
<tr><td rowspan="2">Category 2
Motor function</td><td>Ataxia</td><td>(-)</td><td>Mild (e.g., slow righting response, swaying)</td><td>Moderate (e.g., staggering, impaired walking)</td><td>Severe (e.g., crawling)</td></tr>
<tr><td>Strength</td><td>Decreased strength of lower limbs</td><td>No ability to climb a slope</td><td>No ability to walk</td><td>No ability to right</td></tr>
<tr><td rowspan="2">Category 3
Appearance</td><td>Abnormal posture</td><td>Slight</td><td>Mild (e.g., hunched, extended, low posture, straub tail)</td><td>Moderate (e.g., ventral recumbency)</td><td>Severe (e.g., lateral recumbency)</td></tr>
<tr><td>Abnormal breathing</td><td>Normal</td><td>Normal</td><td>Shallow</td><td>Labored</td></tr>
<tr><td rowspan="2">Category 4
Hyperactivity</td><td>Increased home-cage exploration</td><td>Slightly increased (e.g., increased exploration)</td><td>Increased (e.g., digging, burying)</td><td>Moderately increased (e.g., scratching limbs)</td><td>Marked increased</td></tr>
<tr><td>Stereotype</td><td>(-)</td><td>Slight (e.g., increased grooming)</td><td>Moderate (e.g., circling, repetitive behavior)</td><td>Marked</td></tr>
<tr><td rowspan="2">Category 5
Involuntary movement</td><td>Tremors</td><td>Detectable</td><td>Marked</td><td>N.A.</td><td>N.A.</td></tr>
<tr><td>Seizure (e.g., running, bouncing, clonic and/or tonic seizures)</td><td>(-)</td><td>(-)</td><td>Few or partial</td><td>Repeated or continuous (e.g., >1 min)</td></tr>
<tr><td>Death</td><td colspan="5">Score 22</td></tr>
</table>

Supplementary Table S1. Acute tolerability scoring system

Acute toxicity scoring system used for mouse subjects. The scoring system consists of five neurobehavioral categories: (1) Consciousness, (2) Motor function, (3) Appearance, (4) Hyperactivity, and (5) Involuntary movement. Mice that died during or after i.c.v. administration were assigned a score of 22. N.A., not applicable.

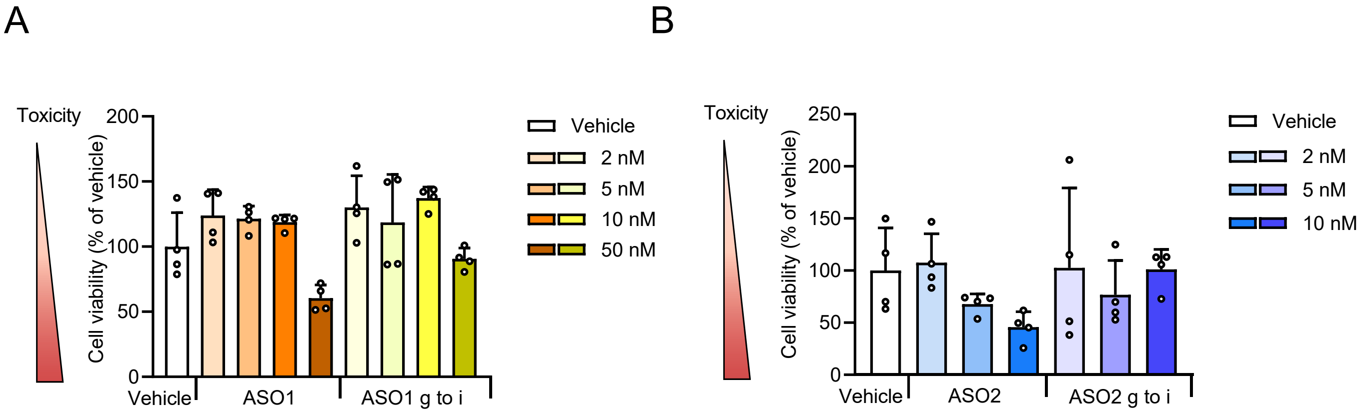

Supplementary Figure S1. Effects of guanine-to-hypoxanthine substitution on ASO cytotoxicity *in vitro*

(A) Cell viability in Neuro-2a cells 48 h after transfection with 2, 5, 10, or 50 nM ASO1, expressed relative to vehicle. (B) Cell viability in Neuro-2a cells 48 h after transfection with 2, 5, or 10 nM ASO2, expressed relative to vehicle. Data are shown as mean ± SEM (n = 4). Statistical differences were examined using a one-way ANOVA followed by Tukey's post hoc test. We observed no significant differences in group means ($p > 0.05$).

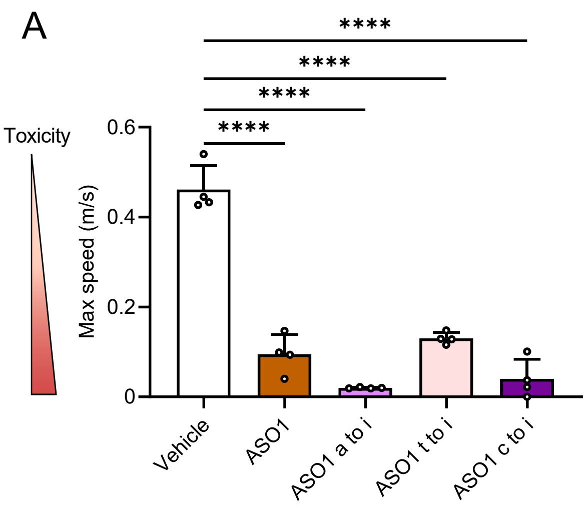

Supplementary Figure S2. Locomotor activity of mice following i.c.v. injection of hypoxanthin-substitution ASOs assessed by open-field test

(A) Locomotor activity of mice assessed after i.c.v. injection of ASO1, ASO1 a to i, t to i, and c to i (9.4 nmol, 50 μg/mouse). Maximum speed at 1 h post-injection in open field tests. Data are shown as mean ± SEM (n = 4). Statistical differences were examined using a one-way ANOVA followed by a Tukey's post hoc test (vs. Vehicle or ASO1; *$p < 0.05$, **$p < 0.01$, ***$p < 0.001$, ****$p < 0.0001$). We observed no significant differences in group means ($p > 0.05$).

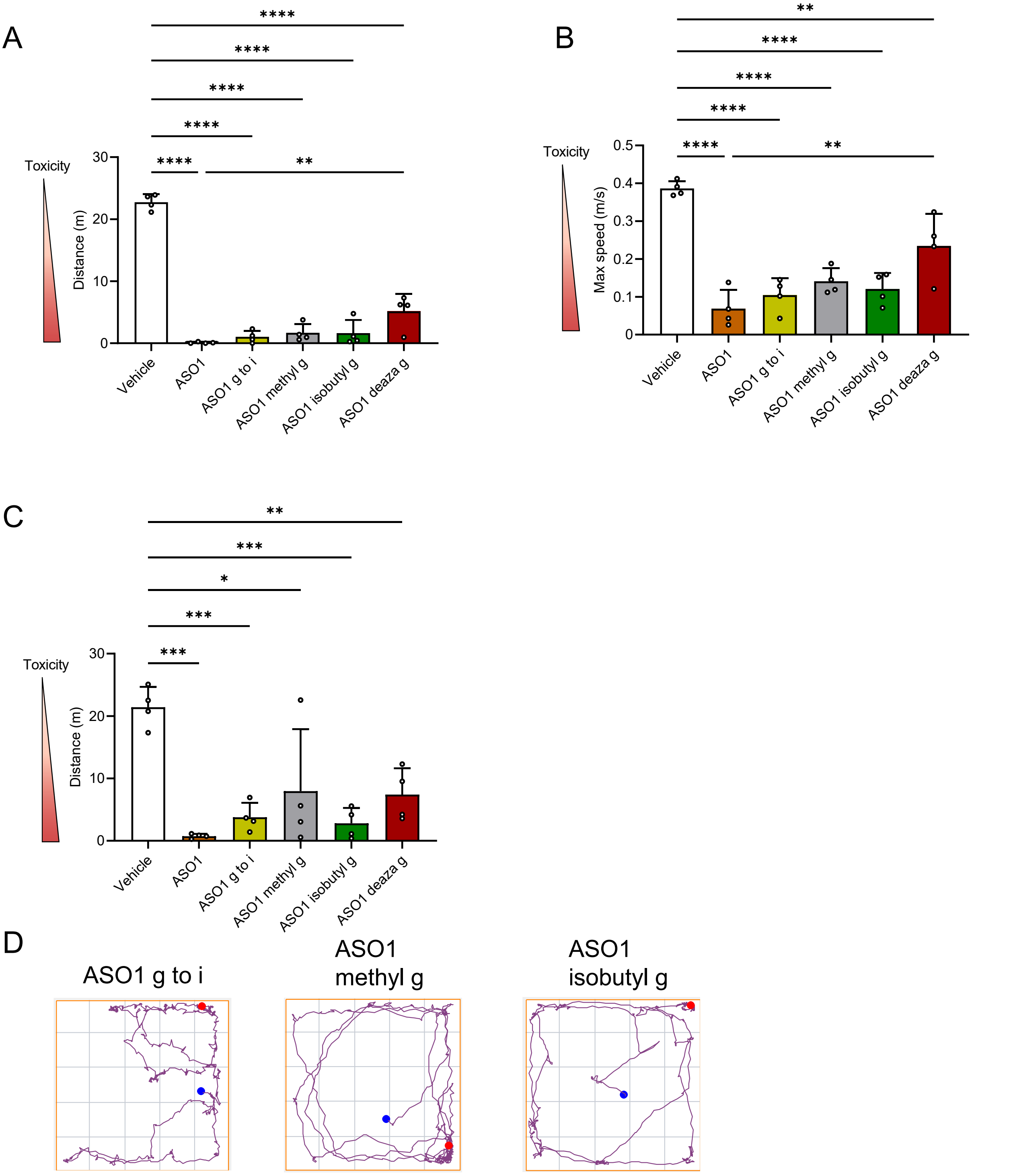

Supplementary Figure S3. Locomotor activity of mice following i.c.v. injection of guanine-modified ASO1s assessed by open-field test

(A–C) Locomotor activity of mice assessed after i.c.v. injection of ASO1, ASO1 g to i, ASO1 methyl g, ASO1 isobutyl g, and ASO1 deaza g (9.4 nmol, 50 μg/mouse). Locomotor activity parameter (A) Represents distance traveled at 1 h post-injection in open-field tests. (B) Maximum speed at 1 h post-injection in open field tests. (C) Distance traveled at 3 h post-injection in open field tests. (D) Representative track plots from open-field tests conducted 3 h post-injection. Data are shown as mean ± SEM (n = 4). Statistical differences were examined using a one-way ANOVA followed by a Tukey's post hoc test (vs. Vehicle or ASO1; *$p < 0.05$, **$p < 0.01$, ***$p < 0.001$, ****$p < 0.0001$). we observed no significant differences in group means ($p > 0.05$).

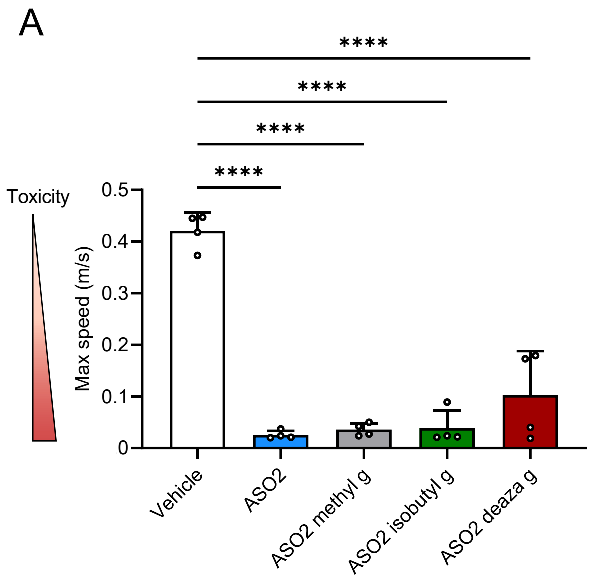

Supplementary Figure S4. Locomotor activity of mice following i.c.v. injection of guanine-modified ASO2s assessed by open-field test

(A) Locomotor activity of mice assessed after i.c.v. injection of ASO2, ASO2 methyl g, ASO2 isobutyl g, and ASO2 deaza g (11 nmol, 50 µg/mouse). Maximum speed at 1 h post-injection in open-field tests. Data are shown as mean ± SEM (n = 4). Statistical differences were examined using a one-way ANOVA followed by Tukey's post hoc test (vs. vehicle or ASO1; **p* < 0.05, ***p* < 0.01, ****p* < 0.001, *****p* < 0.0001). We observed no significant differences in group means (*p* > 0.05).

Supplementary Video S1. Videos demonstrating mitigation of CNS toxicity in mice following i.c.v. injection of ASO

(A) Mouse recorded 1 h after i.c.v. injection of vehicle. (B) Mouse recorded 1 h after i.c.v. injection of 11 nmol ASO1. (C) Mouse recorded 1 h after i.c.v. injection of 11 nmol ASO1 g to i from the experiment shown in Figure 2.

Supplementary Video S2. Videos of mice exhibiting CNS toxicity after i.c.v. injection of ASOs

(A) Mouse recorded 3 h after i.c.v. injection of vehicle. (B) Mouse recorded 3 h after i.c.v. injection of 11 nmol ASO1. (C) Mouse recorded 3 h after i.c.v. injection of 11 nmol ASO1 a to i. (D) Mouse recorded 3 h after i.c.v. injection of 11 nmol ASO1 t to i. (E) Mouse recorded 3 h after i.c.v. injection of 11 nmol ASO1 c to i from the experiment shown in Figure 3.

Supplementary Video S3. Videos showing mitigation of CNS toxicity in mice following i.c.v. injection of guanine-modified ASOs

(A) Mouse recorded 3 h after i.c.v injection of 9.4 nmol ASO1. (B) Mouse recorded 3 h after i.c.v injection of 9.4 nmol ASO1 g to i. (C) Mouse recorded 3 h after i.c.v. injection of 9.4 nmol ASO1 methyl g. (D) Mouse recorded 3 h after i.c.v. injection of 11 nmol ASO1 isobutyl g. (E) Mouse recorded 3 h after i.c.v. injection of 11 nmol ASO1 deaza g from the experiment shown in Figure 4.
